# iPSC-based macrophage models reveal interferon-dependent control of RSV permissiveness and syncytia formation

**DOI:** 10.1128/spectrum.00845-26

**Published:** 2026-07-13

**Authors:** Ayse Agac, Debora Queiros, Eirini Nikolouli, Marie-Christin Knittler, Ariane H. H. Nguyen, Theresa Nägler, Martin Ludlow, Albert D. M. E. Osterhaus, Gesine Hansen, Nico Lachmann, Guus F. Rimmelzwaan, Robert Meineke

**Affiliations:** 1 Research Center for Emerging Infections and Zoonoses, University of Veterinary Medicine Hannover, Hannover, Germany; 2 Department for Pediatric Pneumology, Allergology and Neonatology, Hannover Medical School (MHH), Hannover, Germany; 3 Cluster of Excellence–Resolving Infection Susceptibility, RESIST Hannover Medical School, EXC 2155, Hannover, Germany; 4 Center for Translational and Regenerative Medicine, Hannover Medical School (MHH), Hannover, Germany; 5 Biomedical Research in End-stage and Obstructive Lung Disease Hannover (BREATH), German Center for Lung Research (DZL), Hannover Medical School, Hannover, Germany; 6 Fraunhofer Institute for Toxicology and Experimental Medicine (ITEM), Hannover, Germany; Engelhardt institute of molecular biology, The Russian Federation, Moscow, Russia

**Keywords:** macrophages, iPSC, respiratory syncytial virus, innate immunity, interferons, virus-host interactions

## Abstract

Respiratory syncytial virus (RSV) is a leading cause of severe respiratory illness worldwide and affects mainly infants, immunocompromised individuals, and older adults. Understanding how immune cells contribute to RSV pathogenesis is crucial for developing interventions. However, the lack of model systems that accurately mimic *in vivo* immune responses has hampered progress in this field. Here, we present iPSC-derived macrophages (iMacs) as a promising model for the analysis of tissue-resident macrophage responses during RSV infection. Using monocyte-derived macrophages as a comparison, we show that iMacs are less susceptible to RSV infection. RNA-Seq of iMacs and monocyte-derived macrophages revealed fundamental differences in the baseline expression of immune-related genes between the cell types. Differences in permissiveness were attributed to interferon (IFN) signaling, as a deficiency in IFNAR signaling increased iMac susceptibility to RSV infection, accompanied by increased syncytia formation. These findings present iMacs as a promising research model system for primary macrophages and identify the IFN signaling cascade as a crucial aspect of antiviral responses during RSV infection.

**IMPORTANCE:**

Respiratory syncytial virus (RSV) infections remain a global health burden, with persistent gaps in understanding immune responses that may contribute to disease severity. Early innate immune responses by tissue-resident macrophages are a crucial factor in restricting RSV spread, yet limitations in accessible models have hindered progress in understanding their contribution to protective and harmful immune responses. This study presents iPSC-derived macrophages (iMacs) as a reliable and accessible tool that more accurately replicates the tissue-resident macrophage state compared to monocyte-derived alternatives. Our findings demonstrate the crucial role of effective interferon signaling in limiting susceptibility to RSV infection and the formation of RSV-induced syncytia. Disruptions in interferon signaling increased susceptibility, viral replication, and syncytia formation, revealing key host factors involved in RSV-induced cytopathology. The presented iMac model, therefore, provides deeper insights into protective immune responses, guiding improved therapies and model systems for respiratory virus research.

## INTRODUCTION

Respiratory syncytial virus (RSV) causes significant morbidity and mortality, primarily affecting infants, immunocompromised individuals, and older adults, with more than 33 million cases per year globally (1–3). Although vaccines for prophylactic use in older adults and pregnant women have recently become available, no vaccine has yet been approved for infants, and passive immune prophylaxis of RSV infections relies on the administration of RSV F protein-specific antibodies (4–9). Recent clinical trials in which infant RSV vaccines were discontinued have demonstrated that the pathogenesis remains incompletely understood, despite decades of research (10). Unraveling the dynamics and pathogenesis of infections, particularly during the early stages, may aid the development of effective therapeutic interventions.

Innate immune responses play a decisive role in the early phase of RSV infection. Recognition of viral particles in the extracellular space or sensing within infected cells triggers highly redundant signaling cascades, ultimately leading to the expression and secretion of antiviral cytokines and chemokines that act in both autocrine and paracrine manners (11–13). Type I interferon (IFN) signaling pathways are central to these antiviral responses, allowing the establishment of an antiviral state and the recruitment and activation of immune cells (14). Early and efficient type I IFN secretion by epithelial cells and tissue-resident immune cells is therefore crucial for the local control of respiratory virus infections. Among the first responders to RSV infection are lung-resident macrophages, which produce significant amounts of IFN and are thus important for early antiviral immune responses and for shaping an inflammatory milieu that restricts viral spread (15–17). However, macrophages may also contribute to immunopathogenesis during RSV infection through dysregulated, excessive immune responses (18). Understanding the factors that contribute to a dysfunctional macrophage response during RSV infection is therefore important for dissecting the mechanisms underlying RSV pathogenesis.

Despite their central role in early innate immune responses to RSV infection, research on macrophage responses is sparse, primarily due to limited access to tissue-resident macrophages and the unavailability of appropriate model systems. The isolation of primary human monocytes from peripheral blood mononuclear cells and their subsequent differentiation into macrophages have been the standard for *in vitro* analysis of macrophage responses. However, this model system has several limitations, including donor and batch variations, limited supply, and limited proliferative capacities, which may result in responses that differ significantly from those of primary tissue-resident macrophages. The use of immortalized cell lines, such as THP-1 and U937, may circumvent issues of variability and accessibility; however, immortalized cells differ significantly from primary cells, especially in immune responses (19, 20). An accurate macrophage model is needed to study their role in RSV pathogenesis and immune modulation, to which iPSC-derived macrophages (iMacs) may offer a viable solution.

In recent years, iMacs have emerged as promising tools for macrophage research, offering numerous advantages over conventional *in vitro* approaches (21, 22). Prior studies have confirmed that iMacs match primary macrophages in morphology, gene expression, and functional immune responses, including phagocytosis and IFN signaling (23). Although iMacs are being explored as valuable systems for various infectious diseases and other disease modeling studies, their use in RSV research has been limited.

In the present study, we assessed the suitability of iMacs as a substitute for monocyte-derived macrophages (MDMs) and alveolar macrophage-like (AML) cells in the context of RSV infection. We evaluated their susceptibility to infection and characterized their response to inoculation with a contemporary strain of the RSV A ON1 genotype. Substantial differences were observed between monocyte-derived cells and iMacs, which can be attributed to differences in immune responses, particularly with respect to the IFN gene and ISG expression. Using iMacs deficient in the *IFNAR1* gene (IFNAR^def^ iMacs), we increased permissiveness to RSV infection and observed an adverse effect on syncytia formation in infected cells. The present study, therefore, offers a model for understanding the determinants of macrophage-mediated protective and harmful immune responses in the context of RSV infection. Our findings reveal how IFN signaling modulates RSV permissiveness and syncytia formation, laying the foundation for improved model systems and potential therapeutic strategies.

## MATERIALS AND METHODS

### Induced pluripotent stem cell lines

In this project, the IFNAR1^def^ induced pluripotent stem cell (iPSC) line was generated from human fibroblasts with the mutation Chr21(GRCh37):g.34,726,420_34,728,094del (24) and was used for the generation of iPSC-derived IFNAR1-deficient macrophages. The RUCDRi002-A iPSC line (GMP grade; Lonza, Basel, Switzerland) was used for the generation of IFNAR1-competent macrophages. Generation of the RUCDRi002-A GMP iPSC line was supported by the NIH Common Fund Regenerative Medicine Program and reported in Stem Cell Reports. The NIH Common Fund and the National Center for Advancing Translational Sciences are joint stewards of the RUCDRi002-A resource.

### iPSC culture and differentiation into iPSC-derived macrophages

iPSCs were cultured as feeder-free monolayers in Geltrex-coated (Thermo Fisher Scientific) flasks and DMEM/F-12 (Gibco) supplemented with ascorbic acid 2-phosphate (250 µM, Sigma-Aldrich), sodium selenite (80.9 nM, Sigma-Aldrich), sodium bicarbonate (6.5 mM, Sigma-Aldrich), insulin (3.4 µM, PAN Biotech), and human recombinant transferrin (14.7 nM, Sigma-Aldrich), hereafter described as Essential 6 media (E6), supplemented with TGF-β (80 pM, PeproTech) and bFGF (5.8 nM, PeproTech), from now on described as Essential 8 (E8) media. Media were further supplemented with Y-27632 Rho-kinase inhibitor (10 µM, Tocris Bioscience) for 24 h following cell passaging. Once expanded, the iPSCs were plated for embryoid body (EB) formation. Here, 500 × 10^3^ cells were plated per well of a six-well low-adhesion plate (Greiner Bio-One) in E8 medium supplemented with VEGF (1.3 nM, PeproTech), BMP4 (1.95 nM, PeproTech), SCF (1.1 nM, PeproTech), and Y-27632 Rho-kinase inhibitor (10 µM, Tocris Bioscience) and placed on an orbital shaker for 26 h under 70 revolutions per minute (rpm) agitation. The next day, the media were replaced with E6 medium supplemented with VEGF (1.3 nM, PeproTech), BMP4 (1.95 nM, PeproTech), SCF (1.1 nM, PeproTech), and Y-27632 Rho-kinase inhibitor (10 µM, Tocris Bioscience) for 72 h under 85 rpm agitation. On day 4, the media were replaced with E6 medium supplemented with VEGF (1.3 nM, PeproTech), BMP4 (1.95 nM, PeproTech), SCF (1.1 nM, PeproTech), Y-27632 Rho-kinase inhibitor (10 µM, Tocris Bioscience), and IL-3 (1.67 nM, PeproTech) for an additional 72 h under 85 rpm agitation. At this point, the EBs formed were placed into a new well containing X-Vivo 15 (Lonza), L-glutamine (2 mM, Gibco), penicillin (100 U/mL, Gibco), streptomycin (100 µg/mL, Gibco), 2-mercaptoethanol (0.05 mM, Gibco), IL-3 (1.67 nM, PeproTech), and M-CSF (1.36 nM, PeproTech). X-Vivo differentiation medium was changed every 7 days, and from day 14 onward, EBs began to shed iMacs continuously into the media over several weeks, during which iMacs were harvested weekly for further characterization and/or experiments.

### iMac morphology and surface marker expression

Cytospin slides were prepared to analyze iMac morphology. For this purpose, 4 × 10^4^ cells were resuspended in phosphate-buffered saline (PBS) and centrifuged into SuperFrost microscope slides (Thermo Fisher Scientific) at 600 × *g* for 10 min at room temperature (RT) using a CytoSpin 4 (Thermo Fisher Scientific). Slides were dried at RT and subsequently stained with May-Grünwald staining solution (Roth). Slides were washed thrice with deionized water, followed by Giemsa staining (1:20, Roth) for 20 min. Slides were washed again, dried overnight, and mounted using Histokit mounting solution (Fig. S1A and B). To characterize the surface marker expression and confirm a tissue-resident macrophage phenotype, freshly harvested iMacs were stained in MACS Buffer. Fc-blocking reagent (Miltenyi Biotech) was added to the cells and incubated at RT for 10 min, followed by staining with CD11b-PE-Cy7 (ICRF44, Invitrogen), Tra-1-60R-PE (TRA-1-60-R, BioLegend), CD66b-FITC (G10F5, BioLegend), CD45-ef450 (HI30, Invitrogen), CD14-PE (61D3, Invitrogen), CD163-APC (GHI/61, Invitrogen), CD16-PE-Cy7 (CB16, Invitrogen), or HLA-DR-APC (L243, BioLegend) for 30 min on ice. Surface marker expression was detected using CytoFLEX S (Beckman Coulter) and subsequently analyzed using FlowJo v10 (Fig. S1C and D).

### Generation of MDMs and AMLs

Primary human monocytes were isolated from buffy coat blood provided by the German Red Cross (permit number: 3393-2016) using a two-step gradient-based method. First, peripheral blood mononuclear cells (PBMCs) were isolated by centrifugation with Biocoll (Biochrom) at 460 × *g* for 46 min at RT. After washing the enriched PBMCs with 1× Halt’s buffered salt solution (HBSS) (Gibco), a second density gradient was performed with Percoll solution (Sigma-Aldrich) at 800 × g for 20 min at RT to enrich for primary monocytes. Isolated primary monocytes were washed with DPBS (Gibco) and then cultured in RPMI 1640 medium (Gibco) supplemented with 10% human AB serum (Sigma), penicillin (100 U/mL, Gibco), and streptomycin (100 µg/mL, Gibco) on suspension plates. Human M-CSF (50 ng/mL, PeproTech) was added to the media for the generation of MDMs. The generation of AMLs based on Pahari et al. required the supplementation of 10 ng/mL human GM-CSF, 5 ng/mL TGF-β, and IL-10 (all PeproTech) and 80 µg/mL Curosurf (Chiesi) on days 0, 2, and 4 or, alternatively, on days 0 and 3 with the respective amount (25). After 6 days, prior to harvesting, AMLs are enriched on adherent plates for at least 1 h. Both AMLs and MDMs are then harvested with trypsin. For further experiments, AMLs required a fresh addition of the components used during AML generation.

### Virus stock preparation and infections

All infections were performed using a recombinant (r) RSV-A-0594 strain expressing eGFP (rRSV-A-0594-eGFP), as described previously (26). For virus stock generation, HEp-2 cells (CCL-23, ATCC) were infected at 60%–80% confluency in Opti-MEM (Gibco) supplemented with penicillin (100 U/mL, Gibco) and streptomycin (100 µg/mL, Gibco) and incubated at 37°C and 5% CO_2_. Whole-cell suspensions were collected once cytopathic effects were visible (~3 to 5 days) and centrifuged to separate the cell debris from supernatants (1,000 × *g*, 10 min, 4°C). The clarified supernatants were mixed with 50% polyethylene glycol (PEG) 6000 (Roth) to a final concentration of 10%. The samples were incubated at 4°C for 4 h, centrifuged (3,000 × *g*, 30 min, 4°C), and the precipitated virus was resuspended in HBSS (Gibco) with 20% sucrose. Virus aliquots were snap-frozen in liquid nitrogen and stored at −150°C. Titers were determined in HEp-2 cells by standard 50% tissue culture infectious dose (TCID_50_)/mL assay according to Reed and Muench (27).

For infection of iMacs, IFNAR^def^ iMacs, MDMs, and AMLs, cells were inoculated with rRSV-A-0594-eGFP at a multiplicity of infection (MOI) of 1 and diluted in RPMI 1640 supplemented with 10% fetal bovine serum, penicillin (100 U/mL), streptomycin (100 µg/mL), GlutaMAX (2 mM), 1% MEM vitamins, 1% sodium pyruvate, and 1% nonessential amino acids (R10F, all from Gibco). The cells were incubated at 37°C and 5% CO_2_ for 2 h. The inoculum was then removed, and the cells were washed twice with PBS. The cells were subsequently resuspended in R10F medium and incubated at 37°C and 5% CO_2_ for the duration of the experiment.

### RNA-Seq

Gene expression levels in MDMs, AMLs, iMacs, and IFNAR^def^ iMacs were quantified by RNA-Seq. To this end, cells were either left uninfected or infected with rRSV-A-0594-eGFP (MOI of 1). Total RNA was isolated at 0 h post-infection (hpi, for uninfected samples) or 24 hpi (for infected samples) using a Monarch Spin RNA Isolation Kit (NEB) according to the manufacturer’s instructions. RNA-Seq analysis of mRNA was performed by Novogene via the Illumina NovaSeq X Plus Series (PE150) platform. The data were analyzed using RaNA-Seq and the DESeq2 method (LRT test) (28). The data were visualized by Python (v3.13.5) using Matplotlib and Seaborn libraries. The data presented are based on two biological replicates and two different donors (for MDMs and AMLs).

### Prestimulation and immunofluorescence staining

The cells were pretreated with IFN-α (50 ng/mL, MedChemExpress), IFN-γ (100 ng/mL, Thermo Fisher Scientific), or left untreated for 24 h prior to infection with rRSV-A-0594-eGFP (MOI of 1). After the inoculum was removed, the cells were resuspended in R10F containing IFN-α or IFN-γ, as per the previous stimulus. At 24 hpi, the cells were imaged for GFP expression. For iMacs and IFNAR^def^ iMacs, cells were additionally prepared for immunofluorescence staining by 4% paraformaldehyde fixation at 72 hpi. The cells were then permeabilized with 0.5% Triton X-100 and counterstained with NucBlue (Invitrogen) and ActinRed 555 (Invitrogen) according to the manufacturer’s instructions. All cells were imaged using a Leica DM8 fluorescence microscope.

### Viral replication kinetics

To assess differences in viral replication kinetics between iMacs and IFNAR^def^ iMacs, cells were infected with rRSV-A-0594-eGFP (MOI of 1). Infected cells were incubated at 37°C and 5% CO_2_, and samples were harvested at 0, 24, 48, 72, and 96 hpi. Whole-cell suspensions were collected, and virus particles were precipitated from clarified supernatants using PEG 6000, as described above. The virus was resuspended in HBSS with 20% sucrose and stored at −150°C until titration. Viral titers were determined by standard TCID_50_/mL method in HEp-2 cells, as described by Reed and Muench (27). The final titers were read using a Leica DM8 fluorescence microscope.

### Viral fusion inhibition assay

Inhibition of viral fusion was performed using presatovir (MedChemExpress). Serial twofold dilutions of presatovir (0.2–100 nM) were prepared in R10F. iMacs and IFNAR^def^ iMacs were infected with rRSV-A-0594-eGFP (MOI of 1), and presatovir suspensions were directly added to the cells upon infection. Untreated, infected cells served as a control. The cells were incubated at 37°C and 5% CO_2_ for 72 h. Fusion inhibition was quantified by counting eGFP-positive cells in each well. To this end, three images were randomly captured per well using a Leica DM8 fluorescence microscope. The number of eGFP-positive cells per well was then automatically counted using ImageJ2 (v2.16.0/1.54p).

### Statistical analysis

Statistical analyses were performed using GraphPad Prism (v10.4.1), and the specific statistical tests used are described in the figure legends. For all tests performed, a *P* value of <0.05 was considered significant.

## RESULTS

### Differences in susceptibility to infection and baseline antiviral state

**Fig 1:**
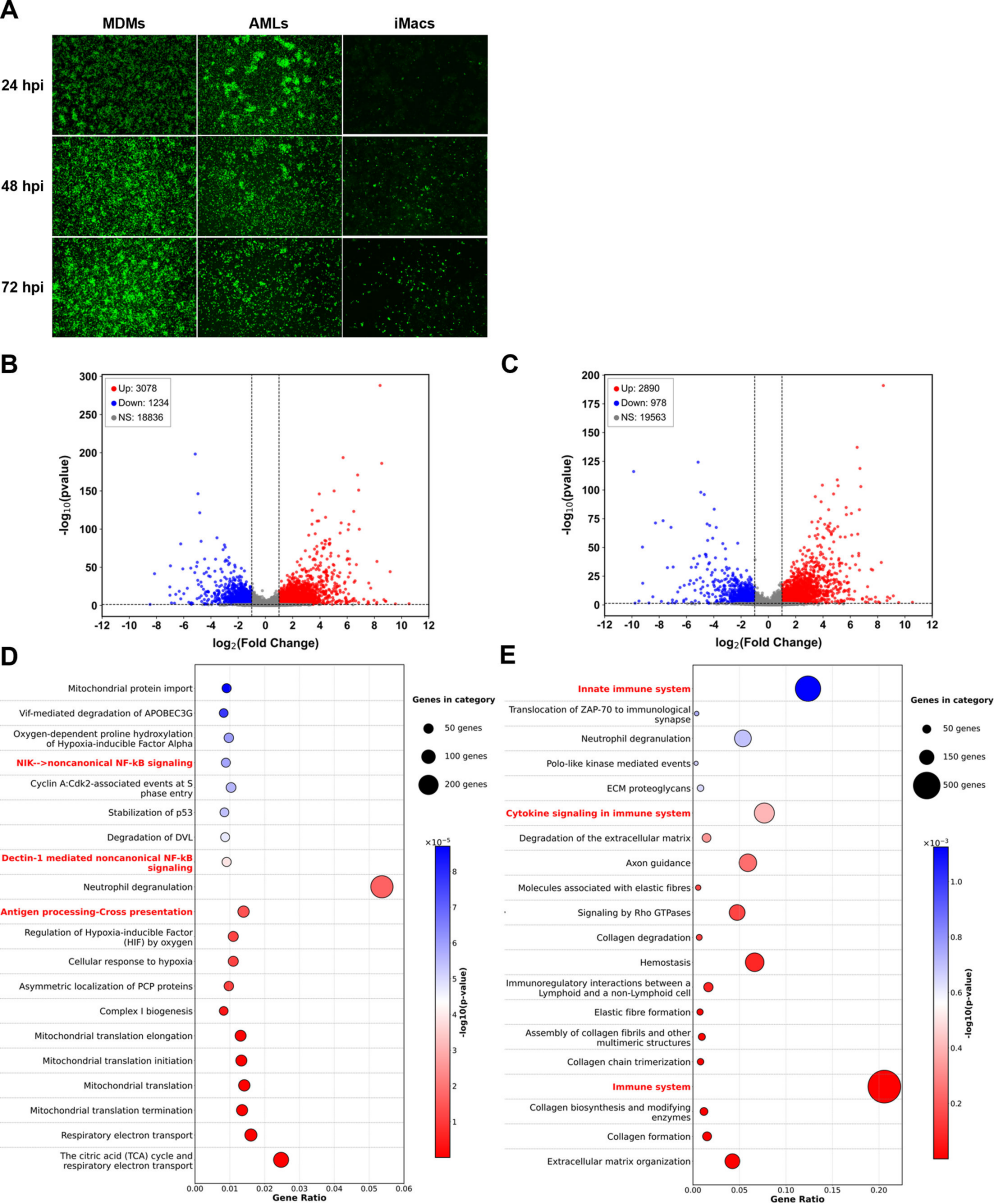
Comparison of monocyte-derived macrophages (MDMs) and alveolar macrophage-like (AML) cells with iPSC-derived macrophages (iMacs). (**A**) Representative fluorescence images of rRSV-A-0594-eGFP-infected (MOI of 1) MDMs (left), AMLs (middle), and iMacs (right) at 24, 48, and 72 hpi. (**B** and **C**) Volcano plots showing the number of DEGs that are upregulated (red), downregulated (blue), or not significantly changed (gray) between MDMs and iMacs (**B**) or AMLs and iMacs (**C**) with thresholds set at *P* < 0.05 and |log_2_FC| ≥ 1 (**D and E**). Bubble plots of the top 20 enriched pathways in uninfected MDMs vs iMacs (**D**) or uninfected AMLs vs iMacs (**E**) based on the REACTOME database. The size of the bubbles indicates the number of genes in the corresponding category. Bubble color corresponds to statistical significance.

To verify that iMacs exhibit a tissue-resident macrophage phenotype and morphology, we performed flow cytometry using surface markers characteristic of this phenotype and May-Grünwald-Giemsa staining, respectively (Fig. S1). The analyzed iMacs expressed CD11b, CD14, and CD45, thereby confirming a myeloid phenotype. Furthermore, expression of the tissue-resident marker CD163 indicates an M2-like macrophage phenotype, whereas the absence of pluripotency and neutrophilic phenotypes was indicated by TRA-1-60R and CD66b, respectively (29). Once the iMac phenotype was confirmed, we continued to determine the permissiveness of MDMs, AMLs, and iMacs to RSV infection. Inoculation with rRSV-A-0594-eGFP, followed by fluorescence microscopic analysis at 24, 48, and 72 hpi, revealed that all cell types were susceptible to infection, as evidenced by the expression of eGFP (Fig. 1A). However, considerable differences were observed between the cell types in terms of the number of eGFP-expressing cells and signal intensity. Specifically, eGFP expression was visible in the majority of the MDMs and AMLs, whereas the signal intensity remained relatively stable throughout the experiment. In contrast, eGFP expression in iMacs was restricted to single cells, with the signal intensity of the individual foci increasing at 72 hpi. No eGFP expression was observed in mock-infected controls for any of the cell types (Fig. S2). We continued to investigate the molecular basis of the observed differences through RNA-Seq analysis, first at baseline levels. Compared with MDMs, iMacs expressed 3,078 genes at higher levels and 1,234 at lower levels (Fig. 1B). The mean log_2_ fold change (95% CI), with a *P* value of <0.05, was determined via the DESeq2 LRT test (Fig. 1B). Compared with those in AMLs, 2,890 genes were upregulated, and 978 genes were downregulated in iMacs (Fig. 1C). Subsequent gene enrichment analysis of differentially expressed genes (DEGs) revealed gene set enrichment in pathways and processes involved in immune signaling (Fig. 1D and E; Fig. S3A and B). Genes associated with these pathways and processes can be classified into clusters based on their function: (i) “cytokines and chemokines” (including genes encoding CCLs, CXCLs, IFNs, and ILs); (ii) “immune signaling receptors” (including genes encoding CCRs, CXCRs, IFN receptors, and IL receptors); (iii) “PRR-dependent signaling cascades” (including genes encoding TLRs, NLRs, IRAKs, and NF-κBs); and (iv) “antigen processing and presentation” (including genes encoding PSMA/B/Cs, HLAs, and TAPs). In clusters 1–3, median expression was significantly lower in MDMs/AMLs compared to iMacs, whereas genes involved in antigen presentation and processing were significantly more highly expressed in MDMs and AMLs than in iMacs.

### Differences in permissiveness leveled by interferon prestimulation

**Fig 2:**
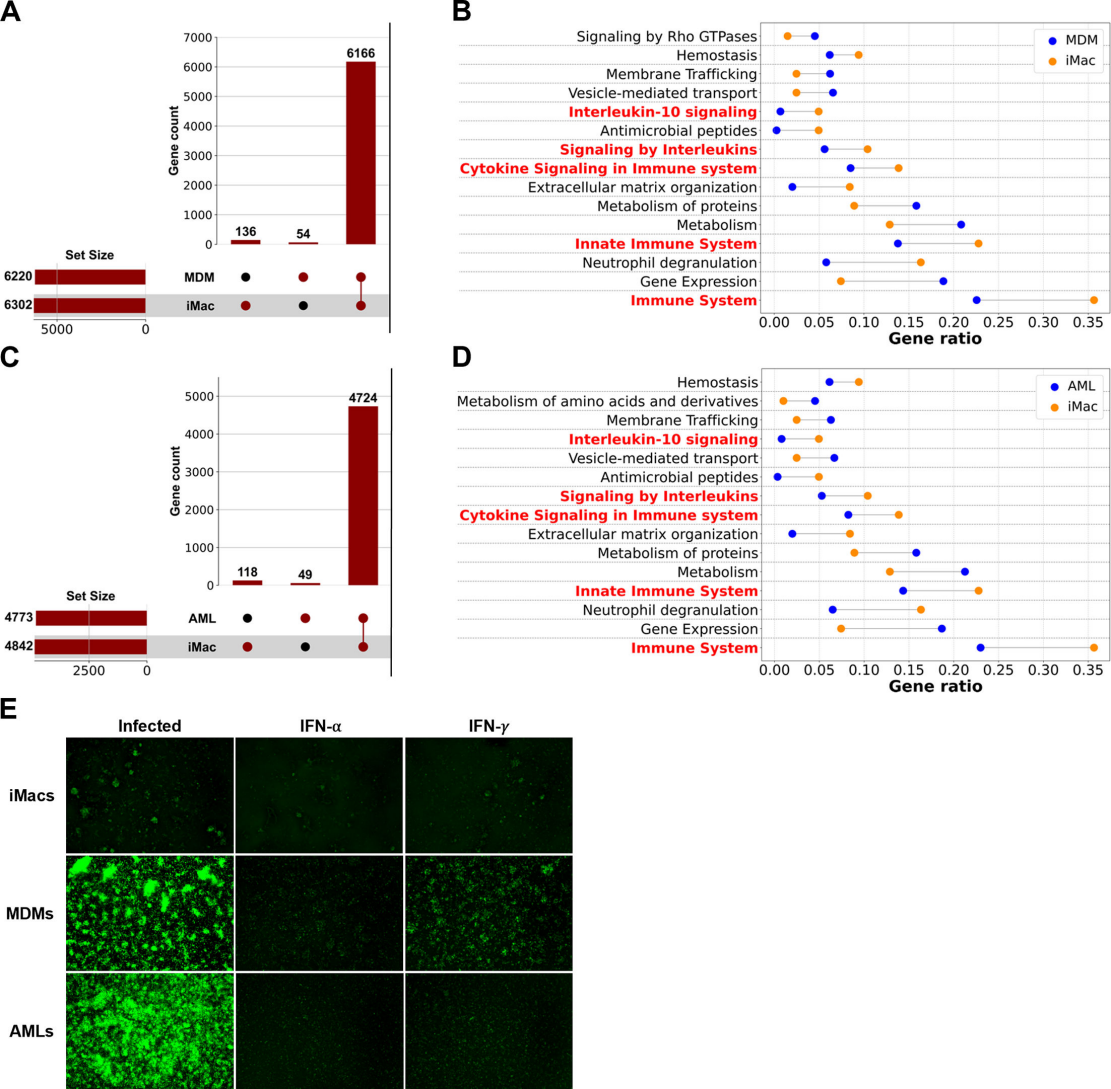
Comparison of MDMs and AMLs to iMacs upon RSV infection. (**A and C)** UpSet plot demonstrating differentially expressed genes that are shared by RSV-infected iMacs and MDMs (**A**), shared by RSV-infected iMacs and AMLs (**C**), or unique to the cell type after comparison to respective uninfected controls with thresholds of *P* < 0.05 and |log_2_FC| ≥ 1. (**B and D**) Cleveland dot plot comparing pathways enriched in both RSV-infected MDMs and iMacs (**B**) or RSV-infected AMLs and iMacs (**D**) based on gene ratios. Pathways are shown in pairs of dots with different colors, representing the compared cell types. The top 15 enriched pathways, which were identified via the REACTOME database, are shown. (**E**) Representative fluorescence images of rRSV-A-0594-eGFP-infected (MOI of 1) iMacs (top), MDMs (middle), or AMLs (bottom) after the cells were pretreated for 24 h prior to infection with 50 ng/mL IFN-α2 or 100 ng/mL IFN-γ or left untreated at 24 hpi.

As significant differences in immune response-related genes were evident at the baseline comparison of MDMs and AMLs to iMacs, we further characterized these differences in the context of infection. To minimize baseline differences independent of viral infection, gene expression upon infection was first compared with that of the respective uninfected control. Pathways and processes enriched upon infection in MDMs and AMLs were compared with those enriched in iMacs, enabling analysis of gene sets commonly shared between cell types. Direct comparisons of DEGs upon RSV infection revealed that the majority of genes were shared between infected MDMs/AMLs and iMacs (Fig. 2A and C). Infected MDMs and iMacs shared more than 6,000 DEGs, while only 136 genes were unique to iMac infection, and 54 genes were unique to MDMs (Fig. 2A). Similarly, more than 4,700 genes were shared between infected AMLs and iMacs (Fig. 2C). Only 118 genes were uniquely differentially expressed in iMacs, and only 49 genes were specific to AML infection. We further dissected the shared DEGs between MDMs/AMLs and iMacs to identify potential differences in gene expression. As expected, RSV infection led to the expression of genes associated primarily with immune responses in all three cell types (Fig. 2B and D and Fig. S4A and B). In both comparisons, the gene ratios in infected iMacs were higher in these pathways and processes than in MDMs or AMLs. Further analysis of the genes assigned to these pathways led to the same categorization as described above, namely, cytokines and chemokines, immune signaling receptors, PRR-dependent signaling cascades, and antigen processing and presentation. The majority of genes assigned to these pathways and processes were significantly upregulated in infected cells compared with uninfected controls for all three cell types. In contrast to the baseline analysis, however, the expression of the genes within all four clusters was significantly greater in infected MDMs and AMLs or comparable to that in infected iMacs. To confirm that immune signaling cascades are responsible for MDM/AML susceptibility to RSV infection, cells were pretreated with IFN-α or IFN-γ before inoculation (Fig. 2E). IFN-pretreated iMacs presented a slight reduction in the eGFP signal upon treatment. Treatment of AMLs with IFN-α or IFN-γ resulted in a decreased eGFP signal, which was comparable to that observed with iMacs. For MDMs, only IFN-α treatment resulted in a reduction comparable to that of iMacs. Although prestimulation of MDMs with IFN-γ reduced the eGFP signal compared with that in untreated cells, the infection remained efficient.

### IFNAR^def^ cells reveal similar susceptibility to infection as AMLs and MDMs

**Fig 3:**
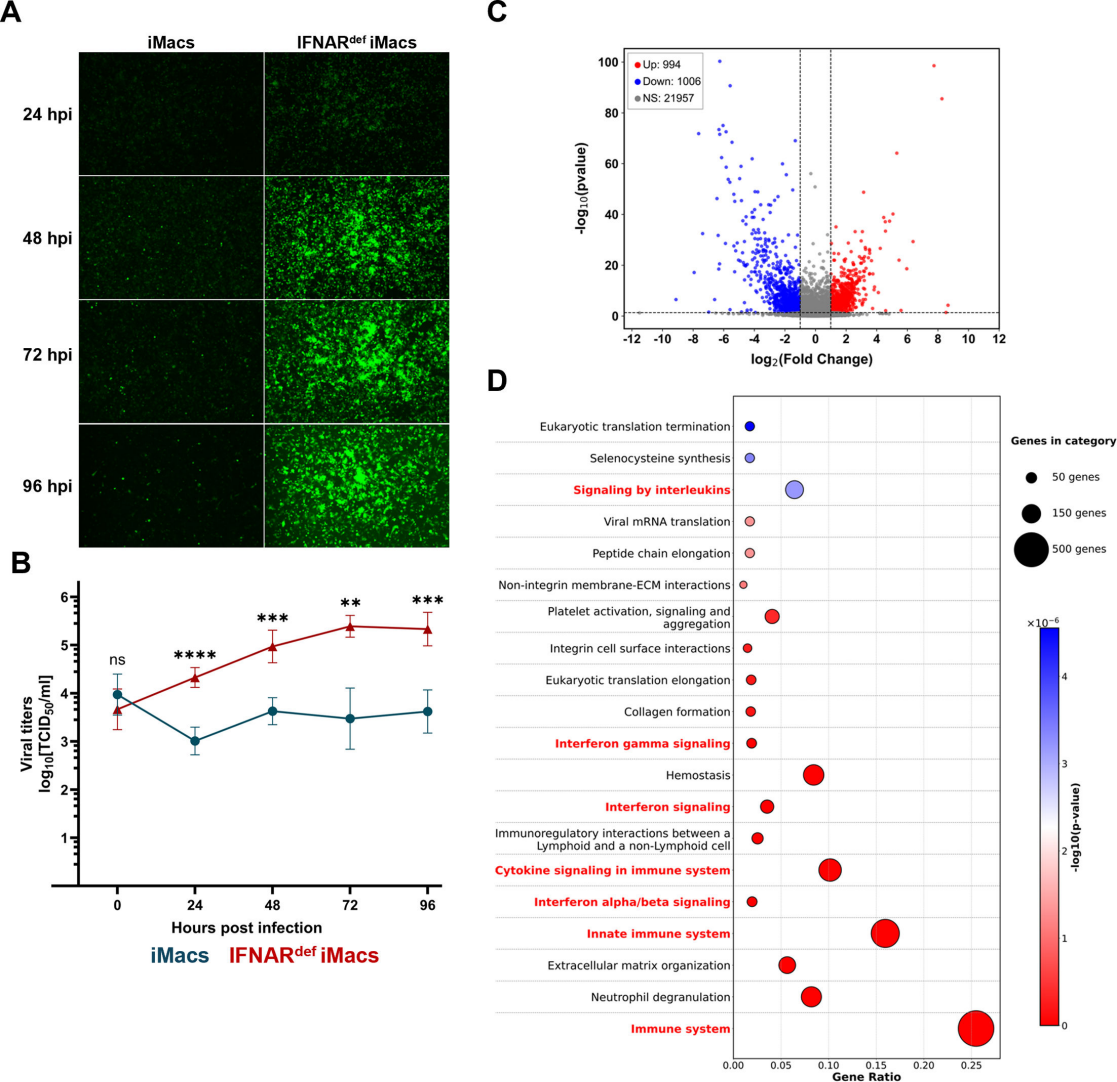
Characteristics of RSV infection in iPSC-derived macrophages (iMacs) with IFNAR deficiency (IFNAR^def^ iMacs) and iMacs. (**A**) Representative fluorescence images of rRSV-A-0594-eGFP-infected (MOI of 1) iMacs or IFNAR^def^ iMacs at 24, 48, 72, and 96 hpi. (**B**) Viral replication kinetics of rRSV-A-0594-eGFP in iMacs (blue) or IFNAR^def^ iMacs (red) at 0, 24, 48, and 72 hpi. The mean ± SD of three independent experiments is shown. Statistical analysis: two-way ANOVA with Tukey’s post hoc test. *P < 0.05, **P < 0.01, ***P < 0.001, ****P < 0.0001. (**C**) Volcano plot showing the number of genes whose expression is upregulated (red), downregulated (blue), or not significantly changed (gray) between iMacs and IFNAR^def^ iMacs under uninfected conditions. Thresholds set at *P*<0.05 and |log_2_FC| ≥ 1. (**D**) Bubble plot of the top 20 enriched pathways in IFNAR^def^ iMacs vs iMacs based on the REACTOME database. Bubble sizes and colors indicate the number of genes associated with the pathway and statistical significance [−log_10_(*P* value)], respectively.

The presented data indicate that immune signaling, particularly IFN signaling, is a fundamental process for susceptibility to infection in macrophages and the key difference between monocyte-derived macrophages and iMacs. We therefore continued to determine the role of efficient IFN signaling in RSV infection of IFNAR^def^ iMacs. Inoculation of IFNAR^def^ iMacs with rRSV-A-0594-eGFP resulted in eGFP expression at 24 hpi, with increasing intensity throughout the experiment (Fig. 3A). Like infected MDMs and AMLs, the majority of IFNAR^def^ iMacs expressed eGFP. The quantification of infectious viral particles secreted from iMacs and IFNAR^def^ iMacs revealed significant differences between the cell types, as RSV titers increased by 1.5-fold upon infection of IFNAR^def^ iMacs (10^3.7^ to 10^5.3^ TCID_50_/mL, 0–96 hpi; *P* = 0.0011) but remained relatively stable for iMacs (10^4^ to 10^3.6^ TCID_50_/mL, 0–96 hpi; *P* = 0.6575), indicating productive infection of IFNAR^def^ iMacs (Fig. 3B). Subsequent RNA-Seq analysis of uninfected iMacs and IFNAR^def^ iMacs revealed 994 genes whose expression was significantly upregulated and 1,006 genes whose expression was significantly downregulated in IFNAR^def^ cells compared with that in iMacs (Fig. 3C). Analysis of enriched pathways and processes revealed that immune responses were different between iMacs and IFNAR^def^ iMacs (Fig. 3D and Fig. S5A). The categorization of these genes into the clusters mentioned above revealed significantly lower expression of most genes within all the clusters (1–4) in RSV-infected IFNAR^def^ iMacs.

### Interferon signaling restricts RSV-induced fusion and syncytia formation

**Fig 4:**
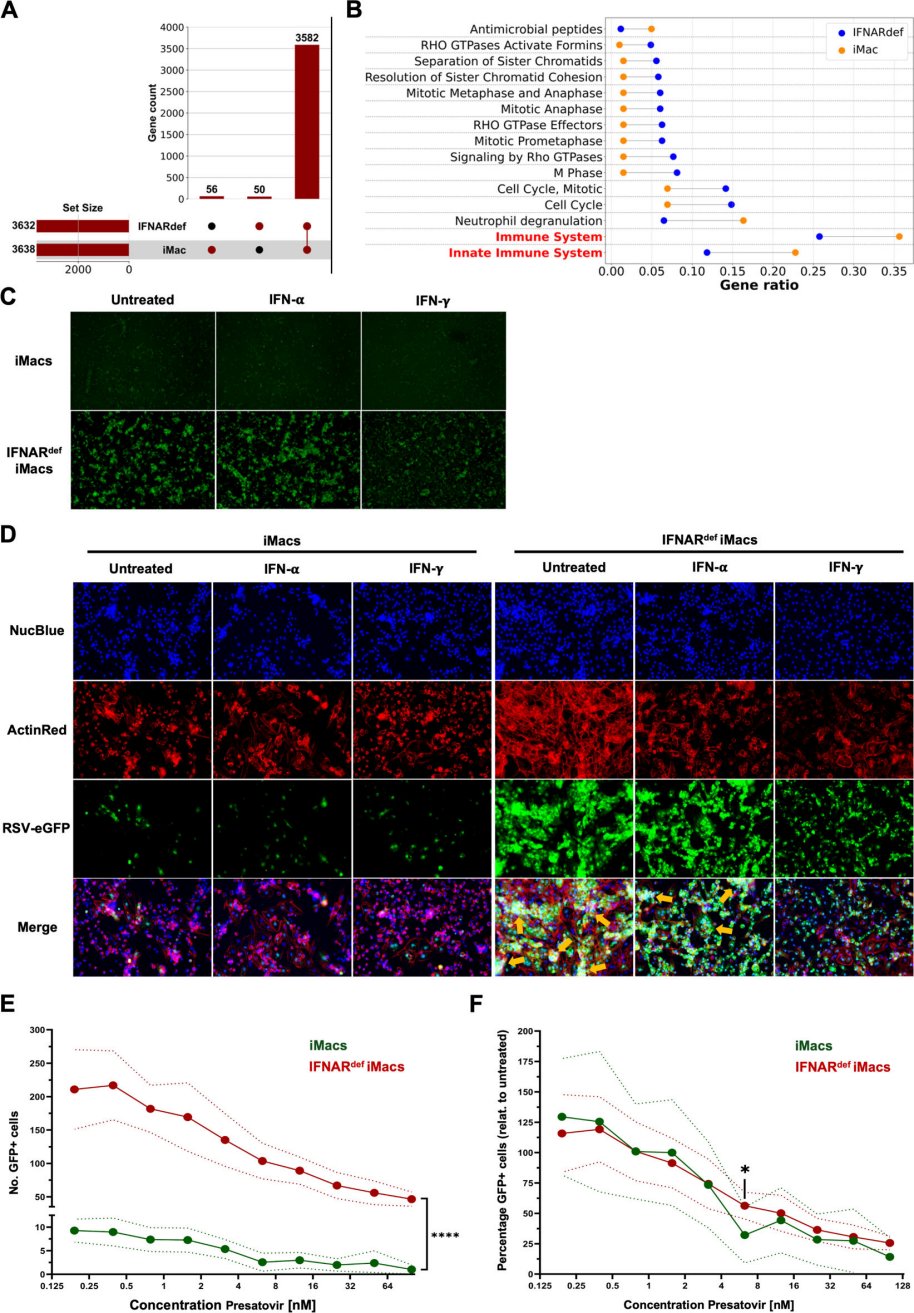
Comparison of rRSV-A-0594-eGFP infection in IFNAR^def^ iMacs and iMacs. (**A**) UpSet plot demonstrating differentially expressed genes that are shared by infected iMacs and IFNAR^def^ iMacs or unique to the cell type after comparison to the respective uninfected controls with thresholds of *P* < 0.05 and |log_2_FC| ≥ 1. (**B**) Cleveland dot plots comparing pathways enriched in both RSV-infected IFNAR^def^ iMacs and RSV-infected iMacs based on gene ratios. Pathways are represented by pairs of dots, enabling direct comparison of gene ratios between the two cell types. The top 15 enriched pathways, which were identified via the REACTOME database, are shown. (**C**) Representative fluorescence images of rRSV-A-0594-eGFP-infected (MOI of 1) iMacs (top) or IFNAR^def^ iMacs (bottom) after the cells were pretreated for 24 h prior to infection with 50 ng/mL IFN-α2 or 100 ng/mL IFN-γ or left untreated. Images were taken at 24 hpi. (**D**) Representative fluorescence images of RSV-infected (MOI of 1) iMacs and IFNAR^def^ iMacs. The cells were pretreated 24 h before infection with 50 ng/mL IFN-α2 or 100 ng/mL IFN-γ or left untreated. The cells were fixed at 72 hpi and stained for nuclei (blue) and actin (red). Multinucleated cells are indicated by orange arrows. (**E and F**) Fusion inhibition assay using presatovir in rRSV-A-0594-eGFP-infected (MOI of 1) iMacs (green) and IFNAR^def^ iMacs (red) at 72 hpi. The graphs show the total number of GFP+ cells (**E**) or the percentage of GFP+ cells relative to untreated, infected controls of the respective cell type (**F**). The mean ± SD of three independent experiments is shown. Statistical analysis for panels **E** and **F**: two-way ANOVA with Šidák’s multiple comparison test. *P < 0.05, **P < 0.01, ***P < 0.001, ****P < 0.0001.

We further characterized the effect of IFNAR deficiency during RSV infection of macrophages. A comparison of the DEGs between infected iMacs and IFNAR^def^ iMacs revealed that the majority (3,852 genes) were shared between the two cell types, whereas 56 and 50 genes were unique to iMacs and IFNAR^def^ iMacs, respectively (Fig. 4A). Analysis of enriched pathways and processes shared between the cell types revealed that immune response pathways were highly relevant, with higher gene ratios observed in iMacs (Fig. 4B and Fig. S5B). Following the categorization of genes into functional clusters, almost all genes assigned to these clusters were significantly downregulated in IFNAR^def^ iMacs. Notably, compared with uninfected controls, both iMacs and IFNAR^def^ iMacs displayed limited upregulation of cytokines, chemokines, and ISGs. To test whether IFNAR^def^ iMacs are responsive to IFN stimulation, cells were pretreated with IFN-α or IFN-γ before inoculation (Fig. 4C and D). As expected, no notable change in eGFP expression was detected after IFN-α prestimulation. In contrast, prestimulation with IFN-γ resulted in a visible decrease in eGFP expression in IFNAR^def^ iMacs. While IFNAR^def^ iMacs were more susceptible to RSV infection, we also observed a phenotypic difference between infected iMacs and IFNAR^def^ iMacs, specifically the apparent formation of multinucleated cells. IFN-γ treatment of IFNAR^def^ iMacs resulted not only in a lower eGFP signal but also in a visible reduction in syncytia formation and size (Fig. 4D). Using presatovir, a small-molecule RSV fusion protein inhibitor that blocks RSV entry into cells, we compared the differences in the efficacy of RSV fusion inhibition between iMacs and IFNAR^def^ iMacs upon infection (Fig. 4E and F). A significantly greater number of eGFP-positive cells was observed for IFNAR^def^ iMacs than for iMacs at all presatovir concentrations tested (*P* < 0.0001) (Fig. 4E). Notably, at the highest presatovir concentration tested (100 nM), eGFP expression was almost undetectable in iMacs (mean_[eGFP+ cells]_ = 1.03 ), while the number of eGFP-positive cells in IFNAR^def^ iMacs (mean_[eGFP+ cells]_ = 46.5) remained even greater than that in untreated iMacs (mean_[eGFP+ cells]_ = 7.8). Normalization of the data to untreated controls revealed no significant differences between iMacs and IFNAR^def^ iMacs, except at 6.25 nM presatovir, where the percentage of eGFP-positive cells was significantly greater in IFNAR^def^ iMacs (56.5% vs 32.2%, *P*=0.0494) (Fig. 4F).

## DISCUSSION

Tissue-resident macrophages represent a critical line of defense during respiratory virus infections, including RSV infection. Given their location within the alveolar space and the lung interstitium, macrophages are among the first cells to respond to infections, restricting virus spread through the phagocytosis of viral particles and the activation and recruitment of immune cells (17, 30). Despite their importance in orchestrating early immune responses, understanding their contribution to protective and harmful immune responses during viral infections has been challenging owing to the limited accessibility of primary, tissue-resident macrophages. This study demonstrated that iMacs are less susceptible to RSV infection than MDMs and AMLs, which is attributed to the higher baseline expression of antiviral genes. Our data show that MDMs (as well as AMLs) are highly permissive to RSV infection, whereas the infection of iMacs is restricted to individual cells. Our findings extend prior research on macrophage polarization and antiviral programming, showing that iMacs maintain a primed antiviral transcriptional profile that restricts RSV entry and replication (31, 32). Using RNA sequencing analysis of MDMs, AMLs, and iMacs, we demonstrated that the high baseline expression of immune-related genes, including various CCLs, IFNs, and interleukins, in iMacs may account for the reduced permissiveness to RSV infection compared with that of MDMs and AMLs. This “primed” transcriptional state of iMacs may reflect the ability of tissue-resident macrophages to enable rapid and effective antiviral responses, thereby restricting viral spread in the early stages of infection. This aligns with the concept of tissue-resident macrophages as immune sentinels, which are involved in the constant surveillance of mucosal tissues and are primed for immediate pathogen sensing and immune responses (33–35).

The pretreatment of MDMs and AMLs with IFNs, which reduces their susceptibility to RSV infection, supports the concept of a primed intrinsic immune state as a protective characteristic of macrophages. These findings also highlight the pivotal role of IFNs in establishing an antiviral state during viral infections. Our results agree with previous findings that identified IFNs as restriction factors for RSV replication through the induction of ISGs (15, 36–38). Importantly, by using IFNAR^def^ iMacs, we were able to dissect the role of IFN signaling in effective macrophage immune responses. The lack of functional IFNAR signaling, and thus type I IFN signaling, increased iMac susceptibility to infection, even allowing for the production of infectious viral progeny. As expected, deficiency in IFNAR signaling was accompanied by a decrease in the expression of immune-related genes upon RSV infection. Notably, our findings revealed a link between IFN signaling and the formation of syncytia in infected macrophages, a hallmark of severe RSV-induced cytopathology (39). These findings therefore suggest that IFN-mediated signaling is a restriction factor not only for viral replication but also for RSV-induced syncytia formation, possibly through the regulation of viral protein synthesis or the establishment of an antiviral state that may alter host properties essential for virus entry and spread (14, 40). Our observations may also be of clinical relevance, as higher concentrations of presatovir, an RSV fusion inhibitor currently in clinical trials (41), are needed to decrease the incidence of IFNAR^def^ iMacs. The findings regarding syncytia formation in IFNAR^def^ iMacs are preliminary, and further *in vivo* studies are necessary to confirm these observations. Considering that the inhibition dynamics were comparable between iMacs and IFNAR^def^ iMacs, differences in fusion inhibition were attributable to cellular properties rather than virus-dependent mechanisms, underscoring the importance of understanding host responses for effective treatment during RSV infection.

While our study offers valuable insights into macrophage responses during RSV infection, the extent to which *in vitro*-generated iMacs recapitulate primary tissue-resident macrophages in a highly complex *in vivo* environment remains unclear. For example, we cannot exclude that differences in differentiation media composition between iMAC and MDM protocols contribute to baseline transcriptional differences and altered STAT1 signaling and ISG expression (42, 43). While no *in vitro* model can accurately replicate the properties of *in vivo* macrophages, our study highlights that the use of different models to address various research questions may contribute to a more comprehensive understanding of macrophage responses during RSV infection. For example, IFNAR^def^ iMacs may provide a valuable tool for understanding the role of different immune responses in restricting syncytia formation and for understanding the molecular basis of RSV cytopathology in at-risk groups.

Taken together, our findings highlight the significant differences in experimental outcomes based on the choice of macrophage model system. iMacs represent a promising model for dissecting early macrophage-driven antiviral responses to RSV. Further research should explore donor diversity, validate findings in primary tissue-resident macrophages, and address the translation of these findings into clinical practice.

## Data Availability

All data sets are available upon reasonable request. Raw RNA-seq data generated in this study are publicly available in the NCBI Sequence Read Archive under BioProject accession number PRJNA1464739.

## SUPPLEMENTAL MATERIAL

Supplemental figures spectrum.00845-26-s0001.pdf Figures S1 to S5.

ASM does not own the copyrights to Supplemental Material that may be linked to, or accessed through, an article. The authors have granted ASM a non-exclusive, world-wide license to publish the Supplemental Material files. Please contact the corresponding author directly for reuse.
