# Supplementary material for: iPSC-based macrophage models reveal interferon-dependent control of RSV permissiveness and syncytia formation
Source: Microbiol Spectr. 2026 Jul 13;14(8):e00845-26. doi: 10.1128/spectrum.00845-26 (PMC13435755; doi:10.1128/spectrum.00845-26)

Supplemental Figure 1: Validation of iMac morphology and surface marker expression. (A+B) Bright-field microscopic images of iMacs **(A)** and $IFNAR^{def}$ iMacs **(B)** after May-Grünwald-Giemsa staining for validation of cell morphology. **(C+D)** Flow cytometric analysis of iMacs **(C)** and $IFNAR^{def}$ iMacs **(D)** for validation of surface marker expression of CD11b, CD14, CD45, CD163, TRA-1-60R, HLA-DR, CD66b, and CD16 gated on live, single cells.

Supplemental Figure 2: Expression of eGFP in mock-infected MDMs, AMLs, and iMacs. Representative fluorescence images of mock-infected MDMs (left), AMLs (middle), and iMacs (right) at 24, 48, and 72 hpi. Images show the absence of eGFP in mock-infected MDMs, AMLs, and iMacs, serving as controls for the eGFP images shown in Figure 1a.

Supplemental Figure 3: Baseline comparison of enriched Gene Ontology terms in MDMs, AMLs, and iMacs. (A+B) Bubble plots showing enriched Gene Ontology terms connected to processes in uninfected MDMs vs iMacs **(A)** or uninfected AMLs vs iMacs **(B)** based on gene ratios. The bubble size indicates the number of genes associated with each GO term, and the bubble color corresponds to statistical significance ($-\log_{10}$(p value)). The top 20 enriched GO terms are shown.

Supplemental Figure 4: Comparison of enriched Gene Ontology terms in MDMs, AMLs, and iMacs upon RSV infection. (A+B) Cleveland dot plot showing the comparison of enriched Gene Ontology (GO) terms connected to processes between RSV-infected MDMs and iMacs **(A)** or RSV-infected AMLs and iMacs **(B)** based on gene ratios. Each GO term is displayed as a pair of dots, allowing for visual comparison between populations, with colors indicating different cell types. The top 15 enriched GO terms are shown.

Supplemental Figure 5: Comparison of enriched Gene Ontology terms between iMacs and $IFNAR^{def}$ iMacs. (A) Bubble plot showing enriched Gene Ontology (GO) terms connected to processes upon comparison of uninfected $IFNAR^{def}$ iMacs to iMacs based on gene ratios. Bubble sizes indicate the number of genes assigned to the GO term, whereas bubble color corresponds to statistical significance ($-\log_{10}$(p value)). The top 20 enriched GO terms are shown. **(B)** Cleveland dot plot showing the comparison of enriched GO terms associated with processes that are shared between RSV-infected iMacs and $IFNAR^{def}$ iMacs. GO terms are represented as a pair of dots, enabling direct comparison of gene ratios between the two populations. The dot color indicates different cell types. The top 15 enriched GO terms are shown.

Supplemental Figure 1:

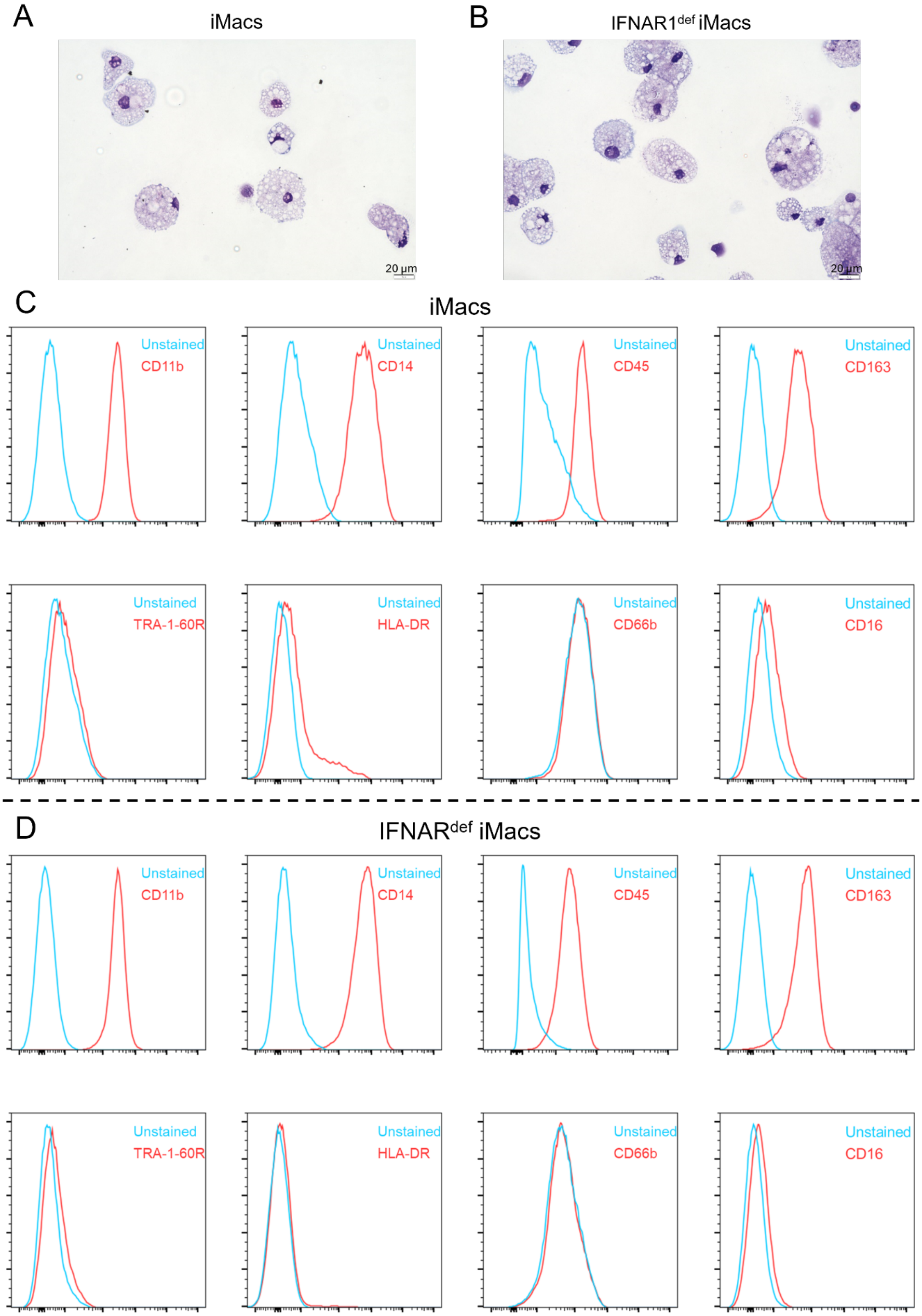

Supplemental Figure 2:

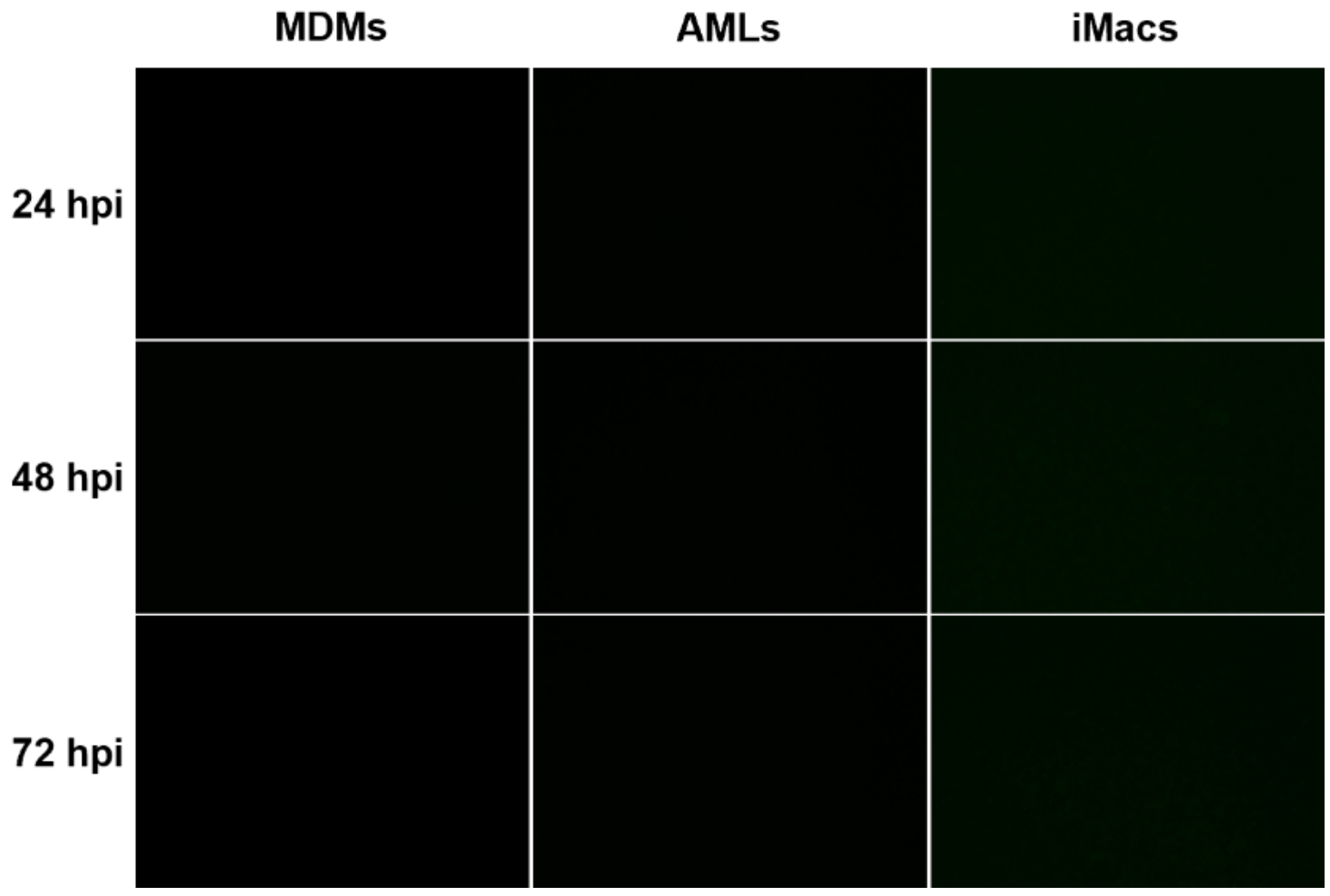

Supplemental Figure 3:

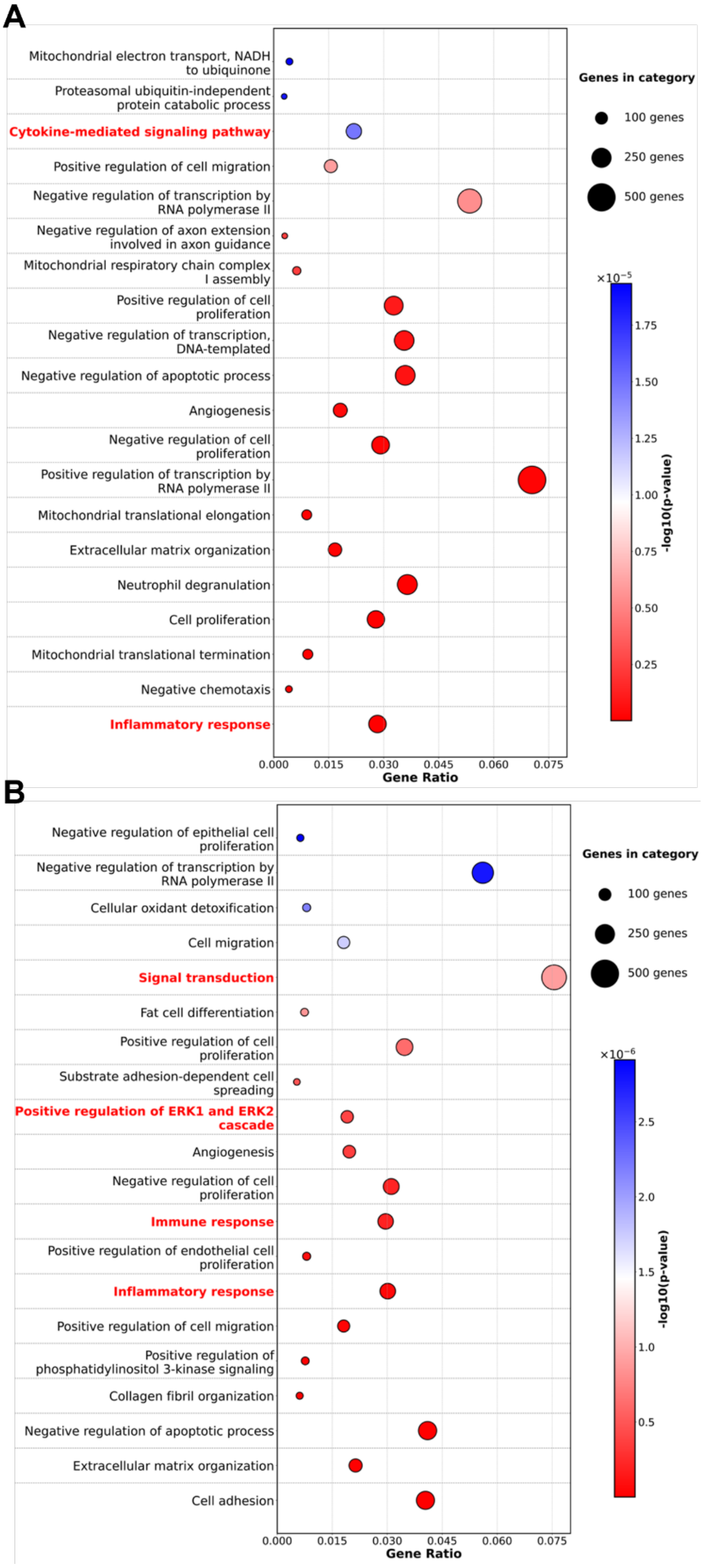

36 **Supplemental Figure 4:**

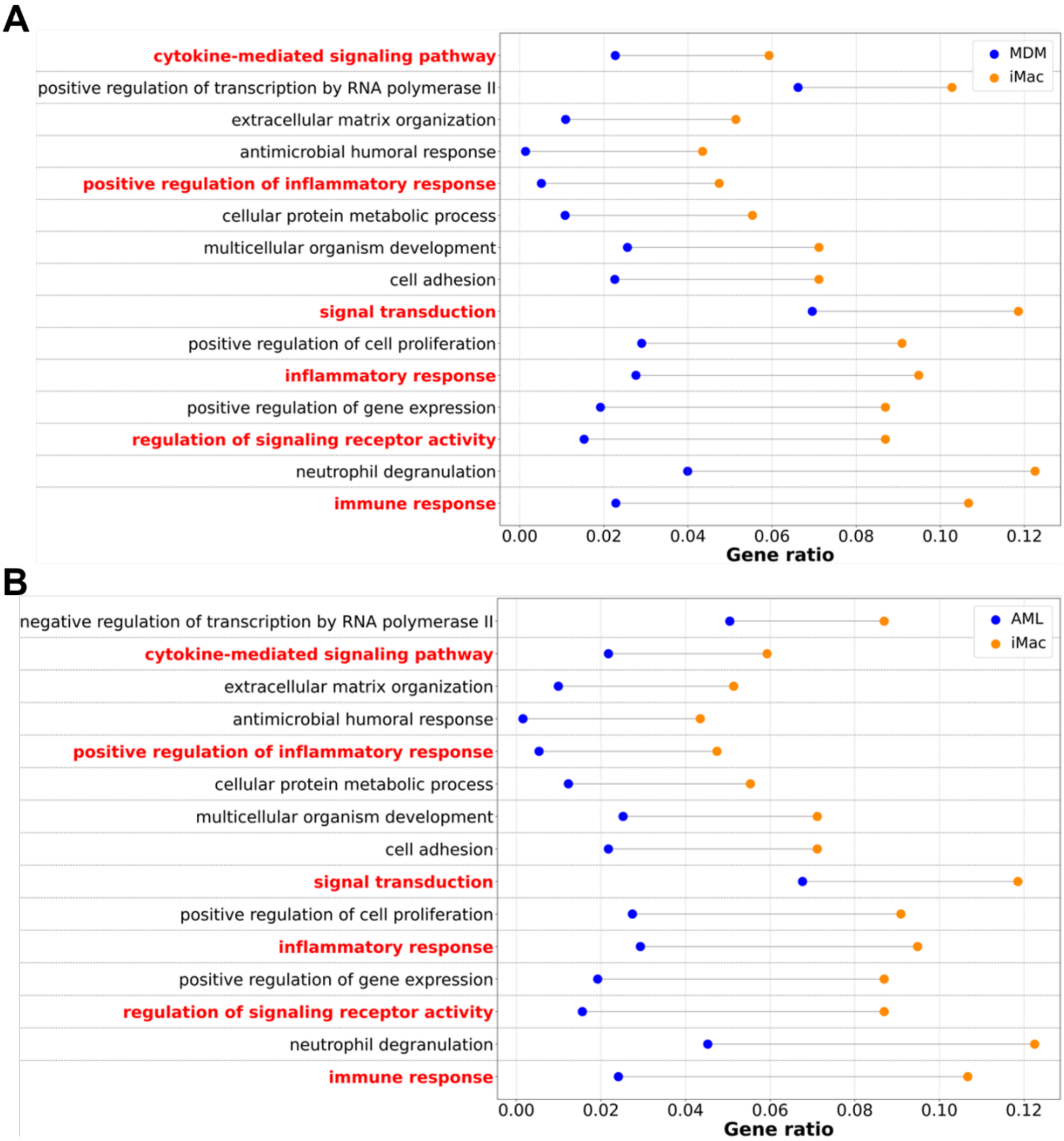

37

Supplemental Figure 5:

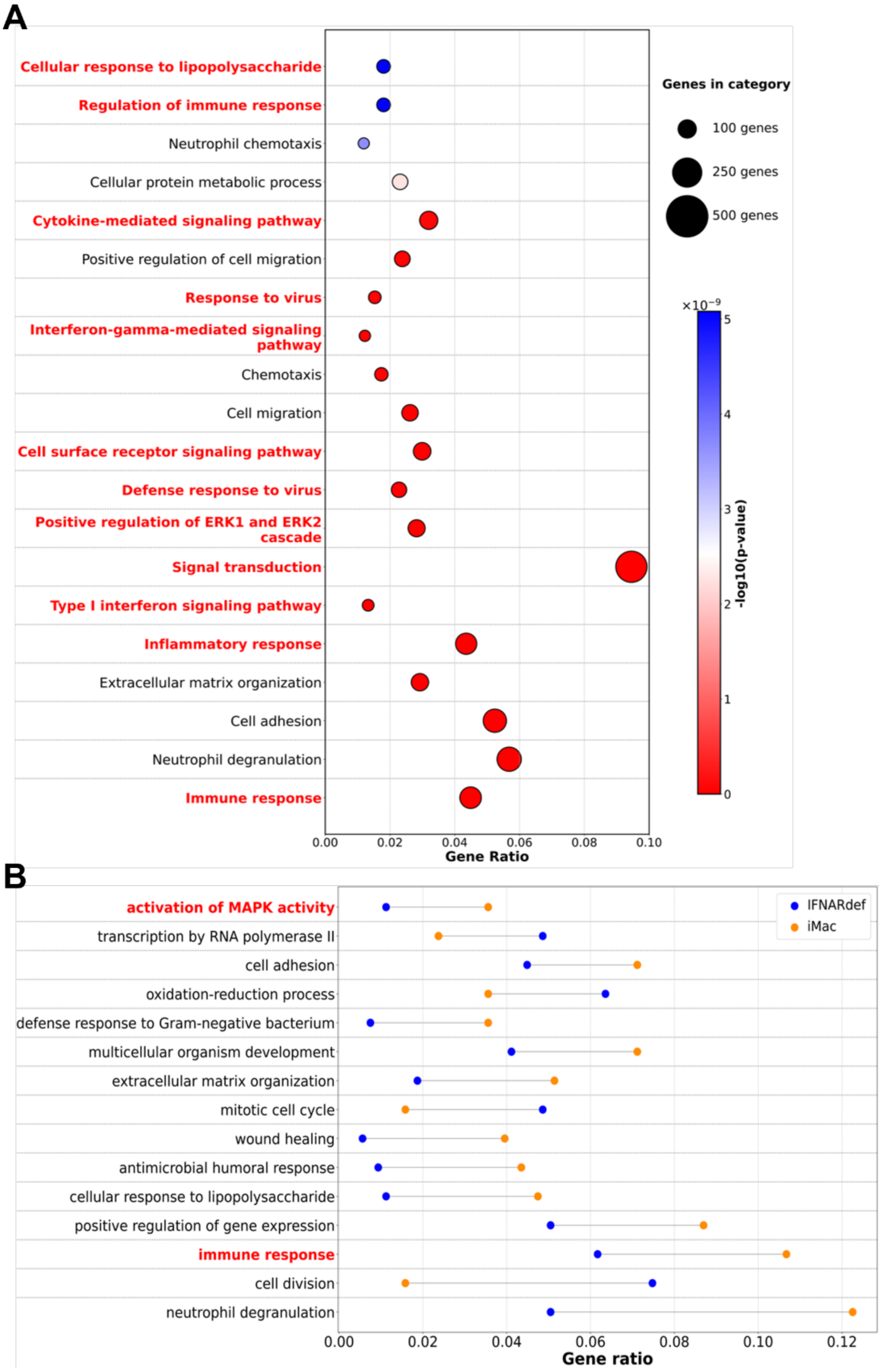

Supplement: Supplementary file 1 [file spectrum.00845-26-s0001.pdf]
